# Targeting Tumor-Associated Macrophages in the Pediatric Sarcoma Tumor Microenvironment

**DOI:** 10.3389/fonc.2020.581107

**Published:** 2020-12-14

**Authors:** Jane Koo, Masanori Hayashi, Michael R. Verneris, Alisa B. Lee-Sherick

**Affiliations:** Department of Pediatric Hematology/Oncology/Bone Marrow Transplant, University of Colorado School of Medicine, Children’s Hospital Colorado, Aurora, CO, United States

**Keywords:** pediatric sarcoma, tumor-associated macrophage, efferocytosis, tumor microenvironment, immunotherapy

## Abstract

For many pediatric sarcoma patients, multi-modal therapy including chemotherapy, radiation, and surgery is sufficient to cure their disease. However, event-free and overall survival rates for patients with more advanced disease are grim, necessitating the development of novel therapeutic approaches. Within many pediatric sarcomas, the normal immune response, including recognition and destruction of cancer cells, is lost due to the highly immune suppressive tumor microenvironment (TME). In this setting, tumor cells evade immune detection and capitalize on the immune suppressed microenvironment, leading to unchecked proliferation and metastasis. Recent preclinical and clinical approaches are aimed at understanding this immune suppressive microenvironment and employing cancer immunotherapy in an attempt to overcome this, by renewing the ability of the immune system to recognize and destroy cancer cells. While there are several factors that drive the attenuation of immune responses in the sarcoma TME, one of the most remarkable are tumor associated macrophage (TAMs). TAMs suppress immune cytolytic function, promote tumor growth and metastases, and are generally associated with a poor prognosis in most pediatric sarcoma subtypes. In this review, we summarize the mechanisms underlying TAM-facilitated immune evasion and tumorigenesis and discuss the potential therapeutic application of TAM-focused drugs in the treatment of pediatric sarcomas.

## Introduction

Pediatric sarcomas are a heterogenous group of tumors that comprise approximately 10% of all childhood cancers (1–5). While sarcomas also occur in adults, the prevalence of subtypes is strikingly unique for the pediatric population. The most common bony pediatric sarcomas are osteosarcoma and Ewing sarcoma (EWS), while rhabdomyosarcoma (RMS) is the most common pediatric soft tissue sarcoma. Other rarer sarcoma subtypes such as synovial sarcoma, leiomyosarcoma, and liposarcomas can occur in children, but are more common in adult patients (6). The cornerstone of treatment typically involves an intensive multi-modality approach including cytotoxic chemotherapy, surgery, and radiation. Over the last five decades, survival improvements have resulted from incremental adjustments to current therapy; however, very few new therapies have been shown to positively improve pediatric sarcomas outcomes (7–9).

For patients with metastatic RMS, the 3-year overall survival (OS) and event-free survival (EFS) are 34 and 27% respectively (10, 11). Survival rates for metastatic osteosarcoma and EWS are similarly dismal with 5-year survival rates reported between 20–30% and 30–40%, respectively (10, 11). Current therapies are highly toxic and associated with many short- and long-term side effects resulting in considerable life-long morbidities (12–15). Alternative approaches, such as immunotherapy, are desperately needed to both improve cure rates and to minimize long-term side effects. Therapeutic approaches that direct the immune system to recognize and destroy tumor cells are currently being trialed in patients with relapsed/refractory sarcomas.

To better understand the potential benefit of immunotherapy in pediatric sarcomas, certain biologic and mutational differences between pediatric and adult sarcomas warrant emphasis. In contrast to adult sarcomas, pediatric sarcomas are generally characterized by a low mutational burden, specific chromosomal translocations that encode “driver mutations,” and low somatic copy number alterations in some sarcoma subtypes (16–23). Higher mutational burden and presence of complex genomic aberrations that occur in adult patients may increase the presence and immune recognition of sarcoma neoantigens. This is further compounded by the observation that the pediatric adaptive immune system tends to be more plastic and may account for variations in individual responses to immunotherapy (24, 25). Additionally, there is higher marrow cellularity and more robust hematopoiesis in children compared to adult patients, exemplified by faster immune reconstitution in children following chemotherapy (26–28). Therefore, these unique differences in sarcoma biology and immune function between adult and pediatric patients likely affect responses to immunotherapy. Furthermore, before cellular immunotherapy can be fully leveraged for pediatric sarcomas an understanding of TAMs within the sarcoma TME is required.

## The Sarcoma Tumor Microenvironment

The cellular composition of the TME is broadly comprised of tumor cells, non-malignant stromal cells, blood vessels, and immune cells. Stromal cells produce extracellular matrix (ECM) proteins and matricellular proteins that provide structural support and mediate signaling for cellular movement. The immune components of the sarcoma TME, including innate immune cells [neutrophils, TAMs, natural killer (NK) cells, dendritic cells (DCs)] and adaptive immune cells (B and T lymphocytes), can vary vastly with respect to sarcoma subtype, primary tumor location, genetic or mutational burden and previous therapy exposure. TAMs are one of at least four myeloid subpopulations derived from tumor-associated myeloid cells (TAMCs) that also include myeloid-derived suppressor cells (MDSCs), tumor-associated neutrophils (TANs), and angiogenic monocytes expressing angiopoietin-2 (TIE-2) (29–31). Cellular immunotherapeutic approaches have largely tested adopted transfer of activated and/or antigen specific T cells; however, efficacy of these cells can be significantly dampened by cells that exert immune regulatory function, including TAMs, regulatory T cells (Tregs), and mesenchymal stem cells (MSCs). For the purposes of this review, we focus on TAMs.

Several studies have demonstrated a strong correlation between macrophage infiltration, sarcoma tumor progression, and patient survival, highlighting TAMs as potential immunotherapeutic targets in pediatric sarcoma (32–36). In addition to phagocytosis of necrotic tumor cells, which decreases the presence of tumor antigen and subsequent immunogenic T cell response, TAMs have been shown to display a wide variety of immunosuppressive and tumor-promoting functions. For instance, increased proportion of TAMs has been shown to render chimeric antigen receptor T cell immunotherapy ineffective (37). However, TAM number and density in pediatric sarcomas do not explain the entirety of their importance in facilitating tumor progression, and the immune cell profiles in pediatric sarcomas vary across tumor subtypes.

## Macrophages in Tumorigenesis

Macrophages play critical roles in innate immunity including: phagocytosis, clearance of apoptotic debris, lymphocyte recruitment (38, 39), antigen presentation (40, 41), wound healing (42), and tissue homeostasis (43, 44). Thus, they both promote inflammatory responses as well as facilitate resolution. In the setting of cancer, macrophages have a response that is seemingly antithetical to the whole organism, as they drive immune tolerance and facilitate cancer progression (45).

Various clinically applicable techniques are in development to identify, quantify, and characterize sarcoma-associated TAMs. Such techniques include immunohistochemistry, single cell RNA sequencing, fluorescent magnetic nanoparticle labeling, and even non-invasive imaging including magnetic resonance imaging (MRI), given that T2* signal enhancement on MR images significantly correlated with TAM density in sarcoma patients (32, 46–48). Depending on their local microenvironments, TAMs can display phenotypic and functional heterogeneity, which is best understood through the concept of macrophage polarization (see next paragraph) (49). However, this dichotomous polarization paradigm is largely oversimplified, and there is a broad range of macrophage polarization phenotypes *in vivo* (50). While TAMs are the largest population of infiltrating immune cells within pediatric sarcomas and TAM infiltration into the tumor can be linked with worse prognosis, the density of TAMs within the tumor does not necessarily provide the full scope of how they influence the TME (34, 51).

## Macrophage Polarization in Tumor Development

**Figure 1:**
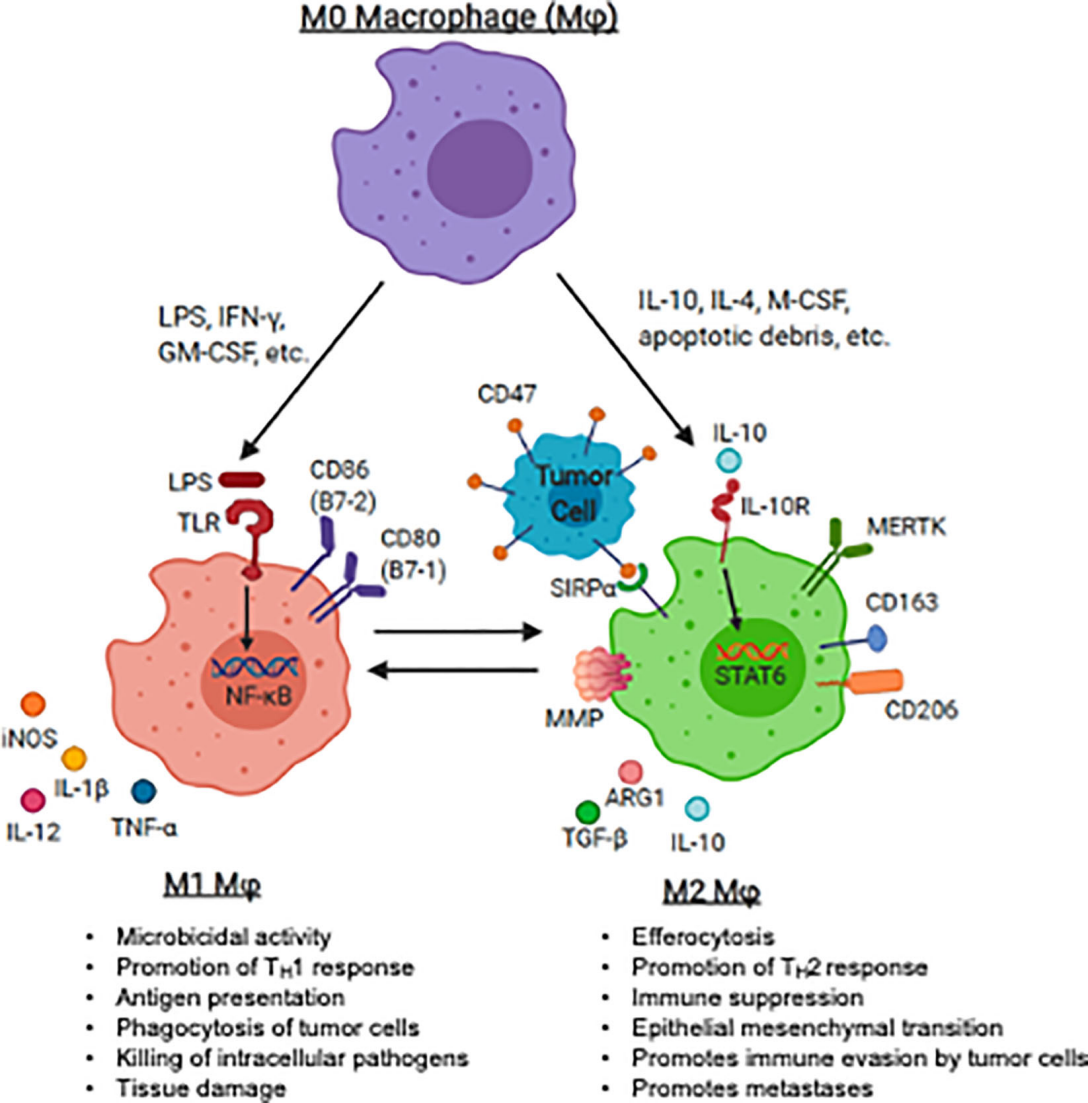
Macrophage polarization and plasticity within the pediatric sarcoma tumor microenvironment. The panel represents recognized M1 (anti-tumoral) and M2 (tumor-promoting) agonists that induce the induction of M1 and M2 markers by human macrophages. The major canonical functions of M1 macrophages and M2 macrophages are also described. LPS, lipopolysaccharide, IFN-*γ*, interferon-gamma; GM-CSF, granulocyte macrophage-colony stimulating factor; IL-4, interleukin 4; IL-10, interleukin-10; IL-13, interleukin 13; M-CSF, macrophage colony stimulating factor; TLR, toll-like receptor; TNF-α, tumor necrosis factor-alpha; IL-1β, interleukin 1 beta; PD-L1, programmed death ligand 1; PD-L2, programmed death ligand 2; MMP, matrix metalloprotease; MERTK, Mer receptor tyrosine kinase; TGF-*β*, transforming growth factor beta. Image created with biorender.com.

The M1/M2 polarization spectrum was developed to explain macrophage phenotype and function in response to inflammation or infection. In the setting of inflammation, M1 macrophages (classically activated macrophages) migrate to sites of infection, phagocytose infected cells and serve as antigen presenting cells (APCs) and produce T helper cell type 1 (Th1) or pro-inflammatory cytokines, promoting T cell activation. In contrast, M2 (alternatively activated) macrophages promote tissue repair through efferocytosis, a phagocytic process in which antigen are cleared, antigen presentation is diminished, and T helper cell type 2 (Th2) cytokines are produced. This process also promotes immune tolerance to autologous (or “self”) tissue. Macrophage plasticity and polarization in the sarcoma TME is also critical for the progression or regression of these tumors (**Figure 1**).

Following exposure to damage- or pathogen-associated molecular patterns (DAMPs or PAMPs), such as bacterial lipopolysaccharides (LPS), nucleic acids, and other microbial ligands, toll-like receptor (TLR) are triggered and M1 polarize macrophage (52–54). TLR ligation initiates a signaling cascade involving the innate immune signal transduction adaptor MYD88, interleukin 1 receptor associated kinase 4 (IRAK4), tumor necrosis factor associated factor 6 (TRAF6) and inhibitor of nuclear factor kappa B kinase subunit beta (IKK-*β*) which ultimately activates nuclear factor kappa B (NF-*κ*B), one of the central regulators of inflammatory cytokine production. Translocation of NF-*κ*B into the nucleus leads to transcription of Th1 genes, such as tumor necrosis factor-α (TNF-α), interleukin (IL)-12, IL-1β, and IL-6, leading to expansion of effector T cells (55–60). Activated T cells produce pro-inflammatory cytokines (*e.g.*, interferon gamma (IFN- *γ*), granulocyte colony-stimulating factor (GM-CSF)) further perpetuating macrophage M1 activation (61–63). Additionally, GM-CSF is a potent driver of antibody-dependent cell-mediated cytotoxicity (ADCC) and antibody-dependent cellular phagocytosis (ADCP), a cell mediated immune defense whereby immune effector cells destroy antibody coated target cells (64–66). M1 macrophages generally express high levels of surface molecules for antigen presentation (e.g., major histocompatibility complex-II (MHC-II)), augment T cell activation (*e.g.*, CD80, CD86), and further promote self-activation (*e.g.*, TLR2, TLR4) (67). M1 macrophages produce cytokines to amplify T cell activation, such as IL-1β, IL-6, IL-12, IL-18 and TNF-α (68, 69).

**Figure 2:**
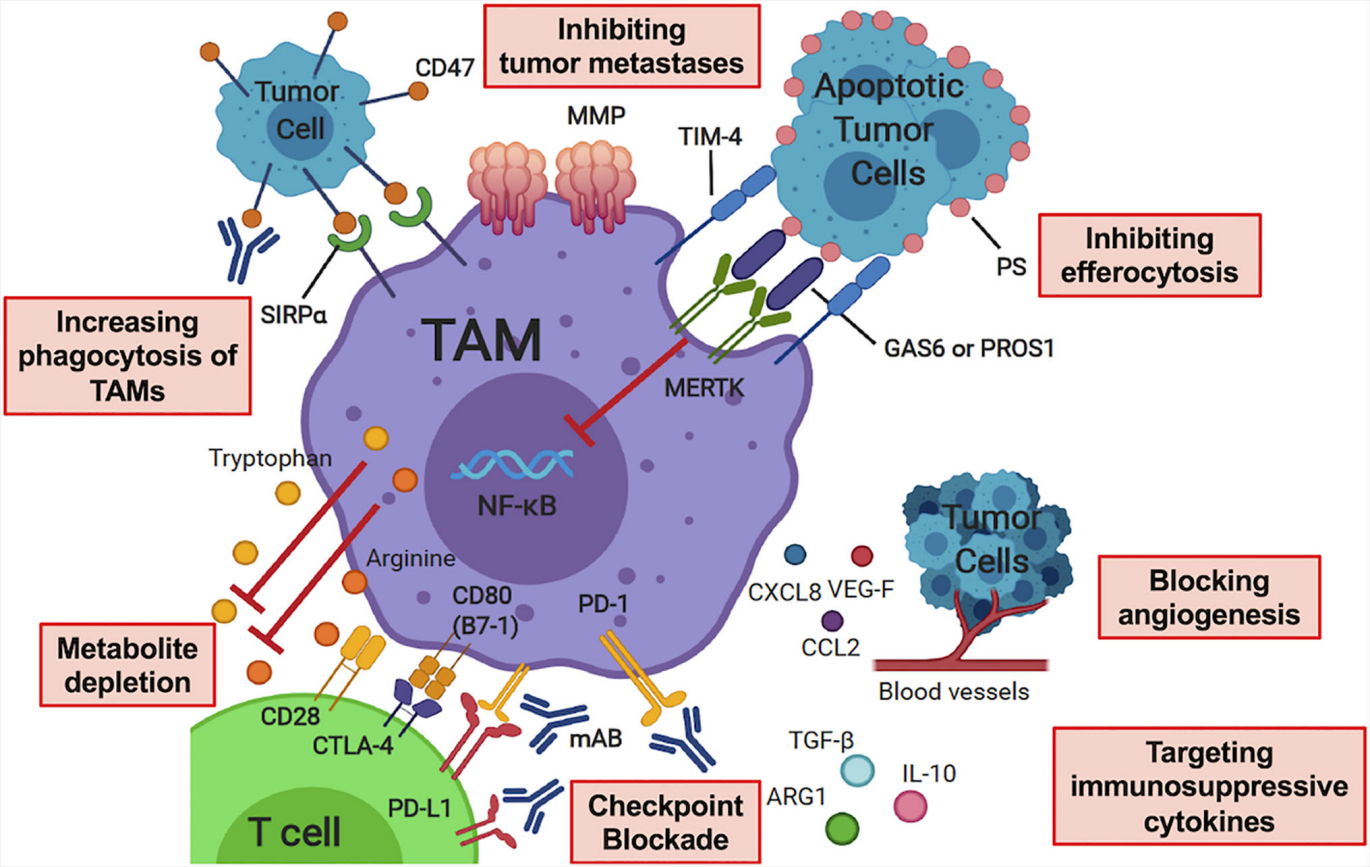
Therapeutic Strategies Targeting Tumor-Associated Macrophages in the Pediatric Sarcoma Microenvironment. Therapy modalities include increasing phagocytosis of TAMs, inhibiting tumor metastases, inhibiting efferocytosis, checkpoint blockade, altering macrophage polarization through targeting immunosuppressive cytokines, metabolite depletion and blocking angiogenesis. TAM, tumor-associated macrophage; SIRP*α*, signal-regulatory protein alpha; MMP, matrix metalloprotease; PS, phosphatidylserine; TIM-4, T Cell Immunoglobulin And Mucin Domain Containing 4; MERTK, Mer receptor tyrosine kinase; PROS1, protein S; GAS6, growth arrest-specific 6; CTLA-4, cytotoxic T-lymphocyte-associated protein 4; PD-1, programmed cell death protein 1; PD-L1, programmed death ligand 1; mAB, monoclonal antibody; IL-10, interleukin 10; TGF-*β*, transforming growth factor beta; ARG1, arginase 1; VEG-F, vascular endothelial growth factor; CXCL8, C-X-C motif chemokine ligand 8; CCL2, C-C motif chemokine ligand 2. Image created with biorender.com

Alternative activation, or M2 polarization, is thought to occur after exposure to cytokines such as IL-4, IL-10, macrophage colony-stimulating factor (M-CSF) and transforming growth factor beta (TGF-*β*) (70, 71), and/or apoptotic cellular debris which promote the resolution of inflammation and wound-healing. M2 macrophages may be identified by the up-regulation of surface markers that promote clearance of apoptotic debris, such as mannose receptor C-type 1 (MMR, CD206) and CD163 (63, 72–76). M2 macrophages may produce T cell suppressive cytokines such as TGF-*β* and IL-10 (77). In response to local cytokine milieu, alternatively activated macrophages also up-regulate inhibitory checkpoint ligands, such as programmed death 1 ligand 1 (PD-L1) and programmed death 1 ligand 2 (PD-L2), which inhibit T cell effector function (78, 79). Many of the above pathways have been or are being considered for targeting to either augment immunity or inhibit the counter-regulatory activity known to occur in malignancy. A summary of therapeutic strategies targeting TAMs in the pediatric sarcoma TME is summarized in **Figure 2**.

### TLR Agonists

Manipulating macrophage polarization in the TME toward M1 activation status has been evaluated using TLR agonists. Muramyl TriPeptide-PhosphatidylEthanolamine encapsulated into liposomes (L-MTP-PE) has been proposed as an adjuvant therapy for osteosarcoma patients. It is a synthetic analog of muramyl dipeptide (MD), a peptidoglycan that is found in bacterial cell walls. L-MTP-PE has been demonstrated to activate TLR4 on macrophages and monocytes and upregulate their tumoricidal functions through increased type 1 cytokine production (such as TNF-α, IL-1, IL-6, IL-8, IL-12, and nitric oxide (NO)) (80, 81). A preclinical evaluation of L-MTP-PE combined with zoledronic acid (ZA) in murine models of osteosarcoma showed that the two drugs significantly inhibited tumor growth and development of metastases (82). Phase I and II clinical trials evaluating L-MTP-PE in pediatric osteosarcoma patients showed acceptable toxicity, and even enhanced macrophage-mediated tumoricidal activity, but had variable results in prolongation of OS and EFS (see **Table 1**) (80, 90, 91). In a follow-up randomized phase III trial [Intergroup (INT)-0133] by the Children’s Oncology Group (COG) for patients with osteosarcoma addition of L-MTP-PE to standard chemotherapy showed no difference in 5-year OS or EFS. When patients with metastatic disease were analyzed separately, L-MTP-PE had improved survival compared (53 *vs* 40%); however, but the study was not powered to detect a significant difference between the two arms (92). L-MTP-PE is not currently approved by the United States Food and Drug Administration (FDA) (102) though the European Medicines Agency granted L-MTP-PE an indication as an adjuvant treatment of osteosarcoma in 2009.

**Table 1:** Current macrophage targeted therapies for the treatment of pediatric sarcomas.

Class	Name	Target	Form	Studies in pediatric sarcoma	Outcomes	Reference
**Cytokines**						
	GM-CSF^1^	Macrophages	Inhaled Inhaled SC^6^	Phase I dose escalation studies in pediatric cancer patients AOST0221: Phase II study of inhaled GM-CSF in first pulmonary recurrence of osteosarcoma patients Phase II study of GM-CSF in combination with chemotherapy and radiation in EWS patients	Limited to no toxicity observed one patient with EWS^2^ achieved a CR^3^; 3-year EFS^4^ and OS^5^ were 7.8 and 35.4%, respectively EFS for 96 patients with osteosarcoma 12% at 4 months; EFS for 42 evaluable patients 20% at 12 months 5-year EFS of non-metastatic EWS patients in group A (with GM-CSF); 0.56 *vs* 5-year EFS in group B (without GM-CSF 0.51. EFS for metastatic EWS was not calculated due to small numbers	(83, 84) (85) (86)
	Zoledronic Acid	Macrophages	IV^7^ IV IV	AOST06P1: Phase I study of ZA^8^ in metastatic OS patients Phase II study of ZA at standard dosing for metastatic osteosarcoma patients EURO-EWING 2012: Phase III randomized, multi-center study combining ZA with standard chemotherapy for EWS patients	DLTs^9^ experienced by five of 24 patients. DLTs included hypophosphatemia, hypokalemia, hyponatremia, mucositis, limb pain and edema; overall EFS and OS for 24 patients were 32% and 60%, respectively Median PFS^10^ 19 months; median OS 56 months among four patients Clinical trial is currently ongoing. (ISRCTN92192408)	(87) (88) (89)
	L-MTP-PE^11^	Macrophages/Monocytes	IV IV	Phase I study of L-MTP-PE in advanced malignancies Phase IIb study of L-MTP-PE in combination with ifosfamide with relapsed osteosarcoma Intergroup-0133: Phase III randomized trial of addition of L-MTP-PE to standard chemotherapy in pediatric patients with metastatic osteosarcoma	Toxicities included fever, chills and hypertension; no major organ-related toxicities observed No increased toxic side effects observed when ifosfamide combined with L-MTP-PE 5-year EFS for patients who received L-MTP-PE *vs* no L-MTP-PE was 46 *vs* 26%, respectively. 5-year OS for patients who received L-MTP-PE vs no L-MTP-PE was 53 and 40%, respectively.	(90) (91) (92)
	Recombinant TNF	Macrophages	IV	Phase I study of rTNF^12^ combined with a fixed dose of actinomycin D in pediatric patients with refractory malignancies	At 240 µg/m2/day of rTNF, three of six patients experienced grade 4 DLT including hypotension, hemorrhagic gastritis, and renal and liver biochemical alterations; antitumor response observed in one metastatic EWS patient	(93)
**Checkpoint inhibitors**						
	Nivolumab	PD-1^13^	IV	Phase II study of nivolumab with or without ipilimumab in patients with unresectable metastatic sarcoma	Clinical trial is currently active not recruiting (NCT02500797).	^-^
	Pembrolizumab	PD-1	IV IV IV	SARC028: phase II study of pembrolizumab assessing safety and activity in patients with advanced soft-tissue or bone sarcomas Phase II study of pembrolizumab and axitinib in patients with advanced alveolar soft part sarcoma and other soft tissue sarcomas PEMBROSARC: Phase II multi-center trial of pembrolizumab with metronomic cyclophosphamide administration in advanced sarcoma patients	Seven (18%) of 40 patients with soft-tissue sarcoma had an objective response two (5%); of 40 patients with bone sarcoma had an objective response including one (5%) of 22 patients with osteosarcoma and one (20%) of five patients with chondrosarcoma. None of the 13 patients with EWS had an objective response. (NCT02301039) Clinical trial is currently active, not recruiting. (NCT02636725) Clinical trial is currently active, recruiting. (NCT02406781)	(94) ^-^ ^-^
	Ipilimumab	PD-1	IV	NCI 08-C-0007: Phase I study of ipilimumab in pediatric patients with recurrent/refractory solid tumors	Immune-related adverse events included pancreatitis, colitis, endocrinopathies and transaminitis. DLTs observed at 5 and 10 mg/kg/dose levels of ipilimumab; one osteosarcoma, one synovial sarcoma and one clear cell sarcoma patient had stable disease for 4–10 cycles. (NCT0144537)	(95)
**Macrophage immunosuppression inhibitors**						
	CB-1158 (INCB00158)	Arginase	IV	Open-label phase I/phase II evaluation of arginase inhibitor INCB00158 as single agent and in combination with pembrolizumab for patients with advanced/metastatic solid tumors	Clinical trial is currently active, recruiting. (NCT02903914)	^-^
**Angiogenesis inhibitors**						
	Bevacizumab	VEG-F	IV IV IV IV IV	Observational off-label study of bevacizumab in combination with cytotoxic chemotherapy salvage and maintenance regimens in pediatric patients with relapsed/refractory sarcomas Phase 1 COG^14^ study of bevacizumab in pediatric patients with refractory solid tumors Phase I study of bevacizumab combined with irinotecan in patients with recurrent, progressive, refractory solid tumors Phase I study of bevacizumab combined with vincristine, irinotecan and temozolomide in pediatric patients with relapsed tumors Phase I study of bevacizumab combined with sorafenib and low-dose cyclophosphamide in pediatric patients with refractory/recurrent solid tumors	Most frequent side effects included epistaxis, transaminitis, acral dermatitis, hypertension, and albuminuria. No DLTs were observed. Non-DLTs included infusion reaction, rash, mucositis, proteinuria, and lymphopenia. DLTs included diarrhea, neutropenia/thrombocytopenia. Maximum-tolerated dose was bevacizumab 10 mg/kg and irinotecan 100 mg/m^2^ DLTs included hyperbilirubinemia and colitis. Other toxicities included diarrhea, hypertension, and myelosuppression. DLTs included rash, lipase elevation, anorexia, and thrombus. Other common toxicities included neutropenia, lymphopenia and rashes.	(96) (97) (98) (99) (100)
**Metastasis inhibitors**						
	Pexidartinib (PLX3397)	CSF1R^15^	IV IV	Phase I/II trial of PLX3397 in pediatric patients with refractory solid tumors and leukemias Phase Ib study of pexidartinib combined with paclitaxel in patients with advanced solid tumors	Clinical trial is currently active, recruiting. (NCT02390752) Adverse events included anemia, neutropenia, fatigue, and hypertension	^-^ (101)

^1^GM-CSF, Granulocyte-macrophage colony stimulating factor.

^2^EWS, Ewing Sarcoma.

^3^CR, Complete response.

^4^EFS, Event-free survival.

^5^OS, Overall survival.

^6^SC, Subcutaneous.

^7^IV, Intravenous.

^8^ZA, Zoledronic acid.

^9^DLT, Dose-limiting toxicity.

^10^PFS, progression-free survival.

^11^L-MTP-PE, Liposomal-Muramyl TriPeptide-PhosphatidylEthanolamine.

^12^rTNF, recombinant TNF.

^13^PD-1, Programmed cell death 1.

^14^COG, Children’s Oncology Group.

^15^CSF1R, Colony stimulating factor 1 receptor.

### Re-Polarizing Agents

Administration of exogenous cytokines to reverse TAM M2 polarization may be an effective immunotherapeutic strategy for pediatric sarcomas. GM-CSF is a myeloid growth factor that stimulates the differentiation of hematopoietic progenitor cells into granulocytes and monocytes with subsequent type 1 cytokine mRNA expression, such as IL-1β, IL-6 and TNF (103). GM-CSF has been successfully incorporated into the standard therapy of high-risk neuroblastoma patients receiving antibody therapy (104). Knowing that the lungs are a common site for pulmonary metastasis, aerosolized GM-CSF has been tested and while it is safe (83–85, 105), it did not improve outcomes for patients with advanced sarcomas (83–85, 105). Similarly, subcutaneous GM-CSF was assessed in a phase II study for 18 pediatric patients with EWS after radiation with no significant difference in 5-year EFS between the treatment group and controls (see **Table 1**) (86).

Alternative methods of delivering intra-tumoral M1 polarizing cytokines have been developed with the goal of minimizing global toxicities associated with exogenous cytokine administration. Innovative methods, such as adoptive transfer of macrophages harboring a soft discoidal particle (“backpack”) that contains the cytokine payload, have been described. In recently published research, phagocytosis-resistant IFN-*γ* secreting macrophage “backpacks” is composed of external polymer layers sandwiching an IFN-*γ* core and a cell-adhesive layer which avidly binds to bone marrow derived macrophages. Adoptive transfer of macrophages carrying IFN-*γ* secreting backpacks into solid tumors maintained their M1 phenotype despite the immunosuppressive TME and also repolarized endogenous M2 TAMs toward an M1 phenotype. This was also associated with decreased tumor volume and lung metastases *in vivo* (106). Further studies into the function, feasibility, and toxicity of these and similar alternative delivery methods are needed as they seem promising as a means of avoiding systemic administration of exogenous cytokines.

## Macrophage Phagocytosis

Efferocytosis is a tolerogenic phagocytotic process characterized by clearance of auto-antigen (or “self”) present on apoptotic cells and suppressing T cell activation. Physiologically, efferocytosis is thought to be critical in the maintenance of self-tolerance and the prevention of autoimmunity. However, in the TME, the otherwise normal TAM process of efferocytosis diminishes immunity through phagocytic clearance of tumor antigen and suppression of T cell cytolytic function, thereby creating a TME supportive of immune evasion and subsequent tumor survival and metastasis. This may be especially relevant in settings of high cell turnover, such as malignancies which are characterized by spontaneous apoptosis due to a myriad of circumstances associated with cancer. Therefore, interfering in the multiple steps involved in efferocytosis may be a novel therapeutic approach with the potential for therapeutic benefit.

### Migration Toward “Find-Me” Signals

Efferocytosis of apoptotic debris is a series of coordinated events, including chemotaxis, recognition and binding of the apoptotic particle, and ingestion. This first step of the sequence includes the secretion of chemoattractant “find-me” signals by a dying cell, including lysophosphatidylcholine (LPC) (107, 108), sphingosine-1-phosphate (S1P) (109), C-X3-C motif chemokine ligand 1 (CX3CL1) (110, 111) and nucleotides (112). Intracellular LPC and S1P are released by apoptotic cells (109, 113), while CX3CL1 is a membrane-associated protein which is cleaved by matrix metalloproteases (MMPs) during inflammation, releasing the soluble protein that acts as a chemokine (114). Nucleotides, specifically adenosine triphosphate (ATP) and uridine-5′-triphosphate (UTP) are released into the extracellular space following caspase-dependent activation. These molecules are recognized by receptors on monocytes and macrophages and result in migration to the area of cellular damage (110, 112, 115). Preclinical studies in RMS and osteosarcoma tumors support the importance of these mechanisms in pediatric sarcoma and have confirmed upregulated expression of bioactive lipids such as S1P, LPC, and lysophosphatidic acid (an LPC cleavage product) in bone marrow extracts (a common site of sarcoma metastasis) by mass spectrometry following radiation and chemotherapy (116, 117). Further clinical studies are required to evaluate the utility of these “find-me” signals as prognostic biomarkers or therapeutic targets for pediatric sarcomas.

### Expression of “Eat-Me” Signals

Tumor cells may evade immune-mediated attack through downregulation of “eat-me” signals. “Eat-me” signals, such as phosphatidylserine (PS) and calreticulin (CRT), are externalized on dying cell surfaces, tagging them for removal by phagocytes. PS is a phospholipid normally localized to the inner membrane of the lipid bilayer in healthy cells; however, during apoptosis, PS accumulates on the cell surface. Similarly, CRT is also exposed on the cell surface during apoptotic stress. CRT interacts with PS and binds the complement C1q protein that serves as both bridging molecule and a PS-binding protein. CRT then binds the SRF-1 endocytic receptor found on macrophages to facilitate phagocytosis of apoptotic cells (118, 119). It is also known that macrophages can utilize their own CRT to enhance phagocytosis of tumor cells (120). Preclinical studies have shown that high expression of PS on EWS tumors increased their sensitivity to tumor necrosis factor-related apoptosis-inducing ligand (TRAIL) mediated cell death (121). Additional studies incubating alveolar and embryonal RMS cells with doxorubicin demonstrated enhanced CRT expression and increased phagocytosis of these RMS cells (4). Other studies are examining the use of “eat-me” signals, specifically CRT, as potential prognostic biomarkers in osteosarcoma (122).

### “Don’t Eat-Me” Receptors

To counter-balance PS or CRT expression, malignant cells may evade macrophage phagocytosis through the expression of “don’t eat-me” receptors. Healthy cells express “don’t eat-me” receptors CD47 and CD31 to avoid unwarranted phagocytic clearance (123, 124). CD47, the prototypical “don’t eat-me” signal, is a membrane protein of the immunoglobulin (Ig) superfamily, present on most cells of the body. Ligation of CD47 with the ssignal regulatory protein alpha (SIRP*α*) protein on macrophages leads to phosphorylation of immunoreceptor tyrosine-based inhibition (ITIM) motifs and a significant inhibitory signaling cascade, characterized by the downstream protooncogene SRC, protein tyrosine phosphatase non-receptor type 6 (PTPN6), and protein tyrosine phosphatase non-receptor type 11 (PTPN11) phosphatases, which inhibit the buildup of myosin-IIA, and prevent the cellular structural changes needed for phagocytosis (2, 125–130). Activation of SIRP*α* has also been found to mediate M2 macrophage polarization, through regulation of the Notch signaling pathway (131–133). Conversely, when the SIRP*α* is blocked, TAMs portend a M1 phenotype (133).

Previous work in experimental models of hematologic and solid malignancies have identified CD47 and SIRP*α* as potential therapeutic targets, whereby blocking this axis (predominantly using anti-CD47 mAb) demonstrated increased phagocytosis of cancer cells by macrophages (2, 4, 5, 126, 128, 134) and M1 polarization (131). Increased phagocytosis of human RMS cells was observed *in vitro* when macrophages were treated with anti-CD47 monoclonal antibody (4). In murine studies of osteosarcoma, CD47 blockade decreased tumor progression, increased macrophage infiltration into the tumor, and increased overall survival (2, 5). Currently, there are no open clinical trials targeting the CD47-SIRP*α* pathway for pediatric sarcomas. However, in adults, there are several open clinical trials evaluating the safety profile and efficacy of anti-CD47 monoclonal antibody (Hu5F9-G4) in a variety of solid and hematologic malignancies (NCT02953509, NCT03248479, NCT03922477, NCT03869190).

### Engulfment and Efferocytosis

Simply put, phagocytosis occurs when the balance of “eat me” signals is greater than the “don’t eat me” signals. Recognition of “eat-me” signals by professional phagocytes occurs through multiple receptors, such as TYRO3, AXL, and MERTK receptor tyrosine kinases and the T-cell immunoglobulin and mucin domain (TIM) receptor family (TIM-3 and TIM-4). Of these, MERTK is the prototypic efferocytosis receptor, given its involvement in the recognition, tethering and engulfment of apoptotic cells, and subsequent generation of immune tolerance through M2 polarization and T cell suppression (135–138). Following apoptotic cell ingestion, MERTK phosphorylation suppresses NF-*κ*B nuclear translocation, leading to diminished type 1 cytokine production (*e.g*. TNF-α and IL-12) (139–141). Conversely, inhibition of MERTK in preclinical studies has shown decreased leukemia-associated macrophage expression of inhibitory checkpoint ligands, including PD-L1 and PD-L2 (discussed below), demonstrating its role in immune tolerance (142). Drugs targeting MERTK have been developed as agents for both reversing cancer progression and cancer immune evasion (143). Pre-clinical studies of MERTK inhibitors in murine solid tumor models have shown decreased tumor growth and increased CTL infiltration (144), while others demonstrated a more profound effect when MerTK inhibition is used in combination with radiation therapy (145).

TYRO3, AXL, and MERTK receptors do not bind to PS directly, rather they use the plasma circulating and locally secreted molecules, protein S (PROS1) and growth arrest specific 6 (GAS6) to provide a bridge to PS. PROS1 binds more specifically to MERTK and GAS6 binds to MERTK, TYRO3, and AXL (146–150). PROS1 and GAS6 are elevated in EWS tumor patient samples, providing increased ligand for efferocytosis to occur (151, 152). Antibodies acting as ligand sinks to bind and inactivate these bridging molecules have been evaluated in preclinical studies but are not yet clinically available (153–155).

TIM family of proteins, TIM-3 and TIM-4, act as PS receptors on macrophages to facilitate the clearance of apoptotic cells (135, 156, 157). On macrophages, TIM-4 works in conjunction with MERTK to mediate tethering and binding of apoptotic cells (156, 158) (159–161). TIM-3, a known co-inhibitory receptor on T cells, is also expressed on antigen presenting cells such as macrophages, aids in the binding and phagocytosis of apoptotic cells through the FG loop in the immunoglobulin variable region (IgV) domain (136, 162–164). Co-expression of TIM-3 with other immune checkpoints such as lymphocyte activating 3 (LAG3) and PD-1 on T cells has been observed in sarcoma patient samples (165); however, its expression on TAMs in sarcoma has not been explored. TIM-3 antibodies are being clinically tested and may be useful in both augmenting T cell activation, as well as diminishing the tolerogenic effects of efferocytosis (166, 167).

Bisphosphonates are a class of drugs designed to inhibit osteoclast activity to prevent loss of bone density in osteoporosis; however, they also suppress macrophage phagocytosis, decrease macrophage recruitment to tumor sites, and increase apoptosis of tumor cells (168, 169). Zoledronic acid is a nitrogen-containing bisphosphate with anti-tumor activity including decreased tumor volume and bone growth in primary EWS tumors, decreased recruitment of TAMs into the tumor stroma in murine sarcoma and carcinoma models (170–172), and reduction in bone metastases EWS after administration of ZA in murine *in vivo* models (171). When combined with ifosfamide, ZA exhibited synergistic effects against tumor growth and progression in a soft tissue tumor model. These promising clinical results have led to the evaluation of ZA in pediatric sarcomas (see **Table 1**). In a phase I study of high-grade metastatic osteosarcoma patients, ZA was well tolerated when administered concurrently with multi-agent chemotherapy (87). One small clinical study evaluated the anti-tumor efficacy of ZA at standard dosing for four patients with advanced stage osteosarcoma with encouraging progression-free survival (PFS) results (88). ZA in combination with standard chemotherapy for EWS patients is currently being evaluated in a multicenter phase III randomized controlled trial (Euro-EWING2012) (89).

## Antigen Presentation

The physical interaction during antigen presentation between macrophage and T cells plays an integral role in T cell-mediated activation and tumor cell cytolysis. Antigen presentation involves three steps, which have been described as different signals. Signal 1 is the binding of peptide-loaded MHC on antigen presenting cells (APCs), such as macrophages, to antigen-specific T cell receptors (TCRs). Signal 2 is the engagement of costimulatory ligands with their cognate receptors on T cells. Conversely, binding of inhibitory ligands (on APCs) with their cognate receptors on T cells inhibits T cell activation. Signal 3 is the secretion of cytokines by APCs which modify or amplify T cell response (165).

### Co-Stimulation and Co-Inhibition

Signal 1, consisting of the MHC-peptide-TCR complex, on its own is insufficient to activate T cells and may generate a tolerogenic response. However, concomitant engagement of adhesion receptors and co-stimulatory ligands (on APCs) and receptors (on T cells), known as Signal 2, creates an immunologic connection between T cells and macrophages (165). Co-stimulatory ligands and cognate receptors are divided into two major groups: CD28/B7 receptor family and TNF/tumor necrosis factor receptor (TNFR) family.

CD28 is a T cell costimulatory receptor that transmits activating intracellular signals when it binds costimulatory ligands CD80 (B7-1) and CD86 (B7-2) on macrophages and other APCs (173, 174). In contrast to the CD28 stimulatory effects on T cells, ligation of inhibitory B7 receptors, including programmed death 1 (PD-1) and cytotoxic T-lymphocyte associated protein 4 (CTLA-4), can promote T cell suppression and/or dysfunction (175, 176). PD-1 has two known B7 ligands on macrophages, including PD-L1 and PD-L2 (177). PD-L1 is often upregulated in tumor infiltrating immune cells including macrophages (178–180). In fact, in pediatric sarcoma patient samples with greater PD-L1 expression, there was higher macrophage and DC infiltration, and a worse outcome (181–183). The treatment of murine and human macrophages with anti-PD-L1 antibodies promotes their proliferation and activation (184). Blockade of the PD-L1/PD-1 axis also enhances macrophage-mediated anti-tumor activity through efferocytosis. Although blockade of this ligand/receptor binding is typically studied for its effects on T cell function, preclinical models of PD-L1/PD-1 blockade using BALB/c *Rag2* ^−/−^ *γc* ^−/−^ mice (which do not have functional T cells) showed TAM-mediated efferocytosis and clearance of tumor cells (185). Disruption of the PD-1/PD-L1 axis in osteosarcoma demonstrated decreased lung metastases, reduced numbers of tumor-promoting TAMs, and increased anti-tumor M1 macrophages in the absence of T cells (183). While anti-PD-L1 or anti-PD-L2 agents have not yet been evaluated in pediatric sarcomas, in a murine model of osteosarcoma nivolumab (an anti-PD-1 monoclonal antibody) increased tumor infiltrating CD4^+^ and CD8^+^ T cells with greater cytotoxic potential (*i.e.*, granzyme B and IFN-*γ* production) and less lung metastases (186). PD-1 blockade using pembrolizumab in the SARC028 phase II study (see **Table 1**; NCT02301039) demonstrated an objective partial response (based on Response Evaluation Criteria in Solid Tumors (RECIST)) in only one osteosarcoma patient out of 22 patients and stable disease in six other patients. Of note, there were no responses in EWS patients, who typically had a low mutational burden (94). Correlative analysis of patient samples from the SARC028 study showed that pembrolizumab responders were more likely to have higher densities of activated CD8^+^CD3^+^PD-1^+^ T cells and increased percentages of PD-L1^+^ TAMs pre-treatment compared to non-responders. Pre-treatment analysis of tumors from responders also demonstrated higher densities of effector memory cytotoxic T cells and regulatory T cells compared to non-responders (187). Given the relatively mutated response of PD-1 axis blockade as monotherapy in pediatric sarcomas, combination strategies with other immune-targeted agents are currently being evaluated in clinical trials (188).

The TNFR family is the other major group of co-stimulatory molecules, which includes CD40, tumor necrosis factor receptor superfamily member 4 (TNFRSF4 or CD134), tumor necrosis factor receptor superfamily member 9 (TNFRSF9 or 4-1BB), and CD27 (165, 189, 190). The co-stimulatory receptor CD40 is a transmembrane protein expressed on monocytes, macrophages, and other antigen presenting cells (191). Its ligand, CD40 ligand (CD40L), is primarily expressed on activated T and B lymphocytes, monocytes and platelets (165). CD40 agonist monoclonal antibodies (mAbs) promote TAM M2 to M1 polarization, leading to increased production of nitric oxide and type 1 cytokines (such as IL-1, IL-12, and TNF-α), and activation of cytotoxic activity of CD8^+^ T cells (192–195). CD40 agonism as monotherapy in advanced solid tumors had limited anti-tumor activity (196); however, use of a CD40 agonist in combination with PD-L1 and CTLA-4 blockade (see below) has shown extended survival in murine solid tumor models (197) (NCT02636725, NCT02332668).

CTLA-4 (also known as CD152) is part of the B7/CD28 family that also inhibits T cell cytotoxic function. CTLA-4 suppresses T cell activation when engaged with its respective ligands, B7-1 (CD80) and B7-2 (CD86) through inhibition of T cell receptor (TCR) signal transduction (198). Sarcoma patients have T cells with high CTLA-4 expression within the tumor and peripheral blood (199, 200). Phase I and II studies of ipilimumab, a CTLA-4 blocking mAbs, in pediatric patients with advanced solid tumors (including sarcomas) showed tolerability but no objective clinical or radiologic responses as monotherapy (NCT01445379) (95, 201). Combination therapies utilizing CTLA-4 and PD-L1 mAb blockade in early phase clinical studies have shown synergistic slowing of disease progression and extended PFS in adults with metastatic or unresectable sarcomas; however, they have not been tested in children (NCT02500797) (202, 203).

### Signal 3: Cytokine Mediated Effects in the TME

Efferocytosis modulates the immune system beyond the regulation of engulfment and co-stimulation in the sarcoma TME. Intracellular signal transduction in efferocytosis favors production of tumor-permissive cytokines such as TGF-*β* and IL-10 consistent with the otherwise physiologic role of this process in immune tolerance, wound healing, and tissue homeostasis (204–206). TGF-*β* drives immunosuppressive responses in both the innate and adaptive immune systems. Within the innate immune system, TGF-*β* secreted by macrophages further skews cells toward an M2 alternative activation status, inhibits cytotoxic and cytokine producing activity of NK cells, and decreases migration and increases apoptosis of dendritic cells (204, 207). In the adaptive immune response, TGF-*β* promotes CD4^+^ T cells to differentiate into Th2 cells and inhibits CD8^+^ T cells antitumor activity by downregulating cytolytic genes such as granzyme B and Fas ligand (FasL), thereby reducing antitumor response (208, 209).

One method of overriding the potently suppressive TAM cytokine production is administration of exogenous type 1 cytokines (210, 211). Interferons and IL-2 are such powerful type 1 stimulators of the immune system. Dinutuximab (ch14.18, a mAb against tumor-associated disialoganglioside GD2) has demonstrated activity against neuroblastoma cells; however, administration of the mAb alone was insufficient to prevent tumor progression, thus both GM-CSF and IL-2 were added to augment efficacy. The addition of IL-2 and GM-CSF to dinutuximab greatly enhanced ADCC by M1 macrophages; however, systemic IL-2 administration was found to have significant toxicity in patients, thus this treatment regimen may still need to be further optimized (104, 212), but could conceptually be applied to other pediatric solid tumors considering the GD2 is also expressed by sarcoma (213, 214).

TNF-α is another type 1 cytokine that has been studied for its effects on augmenting activating antigen presentation within the sarcoma TME. It is produced by classically activated macrophages and lymphocytes and was thought to be a potential immunotherapeutic agent. The majority of exogenous TNF-α administration in preclinical studies was used to mimic chronic inflammation, and thus results were not as favorable as predicted. For instance, a preclinical study of osteosarcoma demonstrated that TNF-α administration promoted the de-differentiation of osteosarcoma cells toward a primitive state, which significantly contributed to tumor growth and progression. Furthermore, blocking TNF-α using a soluble receptor (etanercept) to diminish chronic inflammation inhibited osteosarcoma tumor growth (215). Systemic administration of recombinant TNF-α with chemotherapy in an early Children’s Cancer Group (CCG) phase I study was limited due to systemic toxicities and an inability to dose escalate (93). It has been suggested that further administration of cytokines may need to be targeted to the sarcoma microenvironment rather than systemic administration. As above regarding polarizing macrophages, innovative methods of cytokines delivery will be necessary to allow effective administration without the significant systemic toxicities.

## Metabolism Induced Immunosuppression

Other mechanisms of TAM-induced immunosuppression leading to T cell dysfunction in the TME include breakdown of key metabolites, such as L-arginine and L-tryptophan, which are necessary for T cell activation and proliferation. TAMs produced and secrete arginase 1 (ARG1) and indoleamine 2,3-dioxygenase 1/2 (IDO 1/2), enzymes that catalyze and breakdown L-arginine and L-tryptophan respectively. Breakdown of these metabolites diminishes effector T cell function, thereby increasing the likelihood of cancer cell immune escape (216, 217). In fact, in a study of checkpoint inhibition in adult sarcoma patients where the response rates were lower than expected, the tumor samples had high infiltration of IDO1-expressing TAMs leading to the speculation that elimination of the suppressive TAMs is also needed (NCT02406781) (218). Supplementation with L-arginine in combination with a PD-1/PD-L1 inhibitor in a murine model of both localized and metastatic osteosarcoma increased tumor infiltrating lymphocytes and prolonged survival compared to controls (219). The use of ARG1 targeted small-molecule inhibitors demonstrated reversal of TAM-mediated immunosuppression including production of inflammatory cytokines, CTL, and NK cell tumor infiltration, T cell proliferation, expression of IFN-inducible genes, and restored cytolytic T cell function against solid malignancies *in vitro* and *in vivo* (220). While there are currently no pediatric clinical trials investigating the use of targeted agents against ARG1 and IDO 1/2, there are several studies in adult patients. Additionally, there is an open-label phase 1/2 study investigating an arginase inhibitor (INCB00158) as single or combination therapy with other immune checkpoint therapy in adult patients with advanced/metastatic solid tumors (NCT02903914).

## Tumor Angiogenesis and Metastasis

When tumors reach a certain size, an “angiogenic switch” occurs in which mechanisms are triggered to promote angiogenesis, the formation of high-density vasculature, to increase tumor nutrient supply and improve waste removal (221). TAMs can hasten blood vessel growth through the release of pro-angiogenic factors such as vascular endothelial growth factor (VEGF). Other cytokines released by TAMs such as TGF-*β*, C-C motif chemokine ligand 2 (CCL2), C-X-C motif chemokine ligand 8 (CXCL8), and M-CSF further promote pro-angiogenic functions of macrophages (222–225). On the contrary, M1 polarization of TAMs results in inhibition of angiogenesis through the upregulation of anti-angiogenic factors (such as CXCL8 and IFN-*β*) (226).

VEGF-A is a pro-angiogenic cytokine released by TAMs (227), and has been studied in pediatric sarcomas given that angiogenesis is a critical step in solid tumor progression (228). EWS xenograft models have also showed delayed tumor progression with anti-VEGF directed therapies; however, rebound tumor growth occurred after therapy was discontinued, suggesting single agent VEGF-directed therapy may have limited success in the treatment of pediatric sarcomas (229, 230).

Once angiogenesis has been established, this allows for further tumor progression and metastasis. Metastasis is a complex multi-step process, which starts with tumor cells migrating and intravasating into the vasculature, circulating in the blood stream, eventual extravasation at target organs, and subsequent invasion and growth to establish disease. This complex process requires not only circulating tumor cells, but also requires the close cooperation of perivascular cells, endothelial cells, as well a variety of immune cells including macrophages.

CSF-1 is a chemokine that stimulates macrophage motility/migration, maturation, and survival, and has been implicated in metastasis. Its contribution to metastasis formation was demonstrated in a mammary cancer model where paracrine secretion of CSF-1 by tumor cells stimulated TAMs to migrate and provide a tract for tumors cells to follow along and invade normal tissue and vasculature (223). Congruent with this, immunohistochemistry examination of soft tissue tumor patient samples showed increased expression of CSF-1 (M-CSF) and colony stimulating factor receptor (CSF1R) in more aggressive, higher histologic grade tumors (231). Additionally, CSF-1 mediated mobilization of macrophages and other hematopoietic stem and progenitor cells (HSPCs) are thought to be integral to the formation of the pre-metastatic niche for sarcoma cells at distant sites in the body. In an embryonal RMS murine model, HSPCs were found to be elevated in the peripheral blood during formation of the pre-metastatic niche and contributed to tumor-promoting immunosuppressive myeloid subsets at metastatic sites. Similarly, peripheral blood samples from RMS patients had elevated circulating HSPCs, and patients at greatest risk of metastases had the highest levels of circulating HSPCs at the time of diagnosis (232). Because CSF-1 is essential for TAM migration and maturation, strategic targeting of its receptor (CSF1R) has been explored (233–235). Mice bearing CSF-1 negative neuroblastoma xenografts showed decreased TAM infiltration and angiogenesis, compared to mice with CSF-1 expressing xenografts. Inhibition of CSF1R in neuroblastoma decreased TAM infiltration, improved T cell function, and decreased tumor progression compared to controls (236, 237).

Furthermore, metastasis-associated macrophages (MAMs) are recruited to tumor sites through C-C motif chemokine ligand 2 (CCL2) secretion from tumor cells, a chemokine that mediates monocyte migration from bone marrow to tissue sites through interaction with the macrophage CCL2 receptor, C-C motif chemokine receptor 2 (CCR2) (238). These MAMs secrete additional CCL2 to further augment TAM recruitment to metastatic sites, and CCL3 which instigates tumor seeding at distant sites (239). *In vivo* anti-CCL2 antibody treatment reduced the number of MAMs at metastatic sites and reduced overall tumor burden in breast cancer models (240, 241). Collectively, these studies demonstrate macrophages play roles in the development of metastases and soft tissue infiltration and are potential targets in pediatric sarcomas.

In the clinical setting, attempts have been made to combine therapies targeting tumor angiogenesis and metastasis. For example, bevacizumab (anti-VEGF-A mAb) previously has been combined with conventional chemotherapy backbones, such as vincristine, irinotecan, and temozolamide (VIT), gemcitabine, docetaxel, or low dose cyclophosphamide and sorafenib have shown limited results, producing only stable disease or partial response in a subset of patients with refractory/relapsed disease (see **Table 1**) (96–100, 242, 243).

Additionally for metastasis targeted therapies, there is limited data on combining such drugs with conventional therapies like chemotherapy. Preclinical evaluations demonstrate that combination of CSF1R inhibition after radiation therapy may more effectively decrease tumor volume (244). A majority of clinical trials studying CSF1R inhibitors are in very early clinical trial phases either as monotherapy or combination therapies for the treatment of relapsed/refractory sarcomas. For example, in a phase 1 clinical trial using a CSF1R small molecule inhibitor, pexidartinib (PLX3397) in pediatric patients with refractory solid tumors (including sarcomas) and leukemias showed tolerability, and the expansion cohort is still ongoing (NCT02390752; see **Table 1**) (101). Some trials utilizing monoclonal antibodies directed at CSF-1/CSF1R in adults exhibited limited anti-tumor activity (NCT01346358) (245, 246).

### Chimeric Antigen Receptor Cellular Therapies

The development and clinical use of chimeric antigen receptor (CAR) T cell therapy for the treatment of relapsed/refractory acute lymphoblastic leukemia has provided a promising new therapy option for some patients (247, 248). CAR T cell therapy for pediatric sarcomas has been centered on the development of a CAR directed against GD-2, which is overexpressed on pediatric sarcoma patient samples, with an especially high predominance on osteosarcoma primary and metastatic lesions (249). However, despite the efficacy of CAR therapy in treating hematological malignancies, use of CAR T cell therapy in sarcomas has been more challenging. This is partly due to the difficulty of T cell homing, tumor penetration, and the presence of inhibitory cell subsets in the microenvironment, including infiltrating TAMs that inhibit T cell function.

To overcome such challenges, researchers have explored methods of inhibiting infiltrating suppressor myeloid cells (including TAMs and MDSCs) alongside CAR platforms. In preclinical models, use of all-trans retinoic acid—which differentiated infiltrating myeloid cells, lessening their suppressor function—was found to significantly increase the efficacy of GD-2 directed CAR T cells in pediatric sarcoma models (249). Similar solid tumor models with high levels of TAM or MDSC infiltration found that inhibition of CSF1R increased the efficacy of adoptively transferred T cells (250). As an alternative strategy to mitigate the T cell suppressive effects of TAMs, some groups have engineered their CAR T cells to express cytokines that will lead to TAM M1 repolarization, including IL-12 and IL-18 (251, 252).

Interestingly, the idea of inhibiting TAMs has also been evaluated using a CAR T cell directed against the TAMs themselves. In preclinical models, CAR T cells directed against folate receptor *β* (FR*β*), which is highly expressed by M2 macrophages, lead to cytolysis of M2 macrophages; however, this has not yet been assessed in pediatric sarcomas (253).

Though unrelated to targeting TAMs in pediatric sarcoma, it is notable that some groups are looking into harnessing the infiltrative properties of macrophages by engineering CAR-Macrophages (CAR-Ms). This therapy could be used to direct phagocytic anti-tumor immunity against tumor antigen expressing cells (*e.g.* human epidermal growth factor receptor (HER2), mesothelin) or use alongside CAR T cell therapies to improve T cell penetration into the sarcoma through ECM breakdown (254, 255).

Given the rising interest in cellular therapy to treat malignancies, targeting TAMs and their closely related MDSC populations in the TME will become increasingly important. Similar approaches to inhibiting TAMs in combination with immunotherapy in development include use of bi- and tri-valent T cell engagers (BiTEs, TriTEs) to deplete CD206 and FR*β* expressing TAMs, inhibition of CXCR2 alongside T cell immunotherapy (*e.g.* nivolumab), or TAM repolarization (to an M1 phenotype) using tyrosine kinase inhibitors (256)

Research on TAM-targeting CAR T cells, TAM repolarizing agents or harnessing effector function of CAR-Ms is rapidly evolving. Further work is required to study the potential use of TAM inhibition in conjunction with immunotherapy in sarcomas to further boost anti-tumor immunity.

## Conclusions

It is evident there is remarkable growth in the field of oncologic immunotherapy originating from overall improved understanding of the interaction of cancer cells, TME, and the host immune system. To enhance responses against pediatric sarcomas, new immunotherapy targets and rational combinations of existing immunotherapeutic agents are being investigated. As one of the major components of the TME, TAMs play an intricate role in the regulation of immune suppression within the tumor microenvironment, augmenting angiogenesis, and promoting tumor metastasis formation. All of these are growing areas of research for potential targets in the treatment of pediatric sarcomas. In this review, we discussed the numerous roles TAMs play in driving the immunosuppressive, tumor-promoting environment in the TME, as well as in promoting metastasis, and how this may be reversed in pediatric sarcomas. TAMs are an emerging novel target that has the potential to circumvent immune evasion and hopefully improve survival for pediatric sarcoma patients.

## Author Contributions

JK and AL-S conducted extensive literature review on this subject. JK AL-S, MV, and MH wrote, critically revised and edited the manuscript. All authors contributed to the article and approved the submitted version.

## Funding

This work was supported by the National Institutes of Health (5K08CA222699-03, AL-S), The V Foundation (AL-S), Hyundai Hope on Wheels (MH, MV, and AL-S), the St. Baldrick’s Scholar Award (MH) and the National Pediatric Cancer Foundation Research Grant (MH).

## Conflict of Interest

The authors declare that the research was conducted in the absence of any commercial or financial relationships that could be construed as a potential conflict of interest.
